# Prevalence and Antimicrobial-Resistant *Campylobacter jejuni* and *Campylobacter coli* in Free-Range Chickens in Northwest Ethiopia

**DOI:** 10.4269/ajtmh.24-0578

**Published:** 2025-06-24

**Authors:** Mesfin Worku, Belay Tessema, Getachew Ferede, Linnet Ochieng, Shubisa Abera Leliso, Florence Mutua, Arshnee Moodley, Baye Gelaw, Delia Grace

**Affiliations:** ^1^ Department of Medical Microbiology, School of Biomedical and Laboratory Science, College of Medicine and Health Science, University of Gondar, Gondar, Ethiopia;; ^2^ Institute of Clinical Immunology, Faculty of Medicine, University of Leipzig, Leipzig, Germany;; ^3^ Animal and Human Health Program, International Livestock Research Institute, Nairobi, Kenya;; ^4^ Animal Health Institute, Addis Ababa, Ethiopia;; ^5^ Department of Veterinary and Animal Science, Faculty of Health and Medical Science, University of Copenhagen, Frederiksberg, Denmark;; ^6^ Natural Resource Institute, University of Greenwich, London, United Kingdom

## Abstract

*Campylobacter* enteritis is the most common bacterial foodborne disease in humans. Long-term use of antibiotics in chicken production may result in antimicrobial resistance in *Campylobacter* strains. Information on the antimicrobial resistance profile of *Campylobacter* species among free-range chickens in Ethiopia is scarce. Hence, this study aimed to determine the prevalence and antimicrobial resistance of *Campylobacter jejuni* and *Campylobacter coli* among free-range chickens in Amhara National Regional state, northwest Ethiopia from November 1, 2022 to April 30, 2023. Cloacal swabs were collected from free-range backyard chickens, directly inoculated onto modified charcoal cefoperazone deoxycholate agar, and incubated at reduced O_2_ concentration at 42°C for 48 hours. Suspected colonies were confirmed at the species level using matrix-assisted laser desorption ionization time-of-flight mass spectrometry. The associated factors were analyzed using the Fisher exact test. A *P* <0.05 at 95% CI was considered statistically significant. Among the 286 cloacal samples, 15.0% (*n* = 43/286; CI: 10.2–19.5) were positive for *Campylobacter* species. *C. jejuni* (60.5%) was more frequent than *C. coli* (39.5%). Of the total isolates, 62.8% (*n* = 27/43), 51.2% (*n* = 22/43), and 16.3% (*n* = 7/43) of the *Campylobacter* species were resistant to tetracycline, ciprofloxacin, and erythromycin, respectively. Of the total *Campylobacter* species isolates, 9.3% (*n* = 4/43) were multidrug resistant. *Campylobacter* species resistance to tetracycline and ciprofloxacin was high in general among backyard chickens. Multidrug-resistant *Campylobacter* species were also identified, and they require special attention to prevent the potential dissemination of the strains to humans in the community.

## INTRODUCTION

Foodborne illness poses a significant threat to the global population, ranking alongside major health challenges such as HIV-AIDS, tuberculosis, and malaria. According to the WHO Foodborne Disease Epidemiology Reference Group, approximately 33 million disability-adjusted life years are lost and 420,000 deaths are reported annually among low- and middle-income countries.^1^

The free-range chicken farming practices in Ethiopia significantly contribute to household economies.^2^ According to a report released by the Central Statistical Authority of Ethiopia, the estimated total number of chickens in 2014 was approximately 51 million, with 99% of them being raised using a free-range (backyard production) system.^3^ Although the free-range chicken farming system is known to promote animal welfare,^4^ a report reveals that the incidence of *Campylobacter* occurrence is higher among chickens that are allowed outdoor access than in those that are raised in confined settings.^5^

Chickens serve as reservoirs for *Campylobacter* species, particularly *Campylobacter jejuni* and *Campylobacter coli*, and they excrete these bacteria through their feces.^6^ *Campylobacter* can be spread from free-range poultry to humans through activities that expose owners to the feces of their chickens. Such activities include cleaning the roaming environment and chickens; transmission can also occur through the handling and consumption of contaminated eggs and meat.^7^^–^^10^ *Campylobacter* enteritis is the most prevalent bacterial foodborne diarrheal disease in human.^11^ *Campylobacter* species have also been associated with rare postinfection complications, like Guillain–Barré syndrome, irritable bowel syndrome, and reactive arthritis.^12^^–^^15^

In the majority of cases, *Campylobacter* enteritis is a self-limiting infection; however, antibiotics may be required for individuals with compromised immunity, particularly those in the younger than 5-years-old and older than 65-years-old age groups, as well as individuals with underlying conditions, such as AIDS, diabetes, and cancer.^16^^,^^17^ The three recommended antimicrobial agents that have been used in human *Campylobacter* enteritis are macrolides (erythromycin), fluoroquinolones (ciprofloxacin), and tetracycline. Although tetracycline is recommenced for alternative use in human campylobacteriosis, it is not in use practically.^18^

Information on antimicrobial resistance of *Campylobacter* species among free-range backyard chickens is scarce in Ethiopia. Therefore, the current study aimed to assess the magnitude and antimicrobial resistance of *C. jejuni* and *C. coli* among free-range chickens.

## MATERIALS AND METHODS

### Study design and study area.

This community-based cross-sectional study was conducted in the urban and periurban areas of Gondar and Bahir Dar cities, northwest Ethiopia from November 2022 to April 2023. Gondar city is an administrative center of the Central Gondar Zone located 657 km away from Addis Ababa, the capital city of Ethiopia. It was estimated that about half a million people live in Gondar city.^19^ Bahir Dar is the capital city of the Amhara National Regional State, which 488 km away from Addis Ababa, the capital city of Ethiopia. According to the Bahir Dar health office, the total population of Bahir Dar was estimated around 267,350.

### Study population, sample size, and sampling techniques.

The sample size was determined using the single-population proportion formula to estimate the prevalence of *Campylobacter* species among backyard chickens. The sample size was determined by using 96.9% prevalence of *Campylobacter* species among chickens,^20^ 95% CIs, and precision of 5%. The sample size was 286 for free-range chickens. The Gondar and Bahr Dar sites were randomly selected. In addition, kebeles (the smallest administrative units in Ethiopia) were also randomly selected from the two sites. The pooled cloacal swab samples were collected from backyard chickens belonging to conveniently selected households located in the kebeles (Weleka, Maraki, Azezo, Abay Mado, Zenzelima, and Shimbit). The household head or another household member 18 years old or older was interviewed about the reproduction conditions of the free-range chickens.

### Collection, transportation, and processing of cloacal swabs.

The procedure was strictly followed as previously described; in brief, cloacal swabs were obtained by carefully inserting a sterile cotton swab to the appropriate depth of the cloaca. Once the samples were collected, they were placed into universal bottles containing Cary-Blair Transport Medium (Oxoid, Thermo Fischer Scientific, Basingstoke, Hampshire, United Kingdom). Then, the specimens were kept in an ice box containing ice packs with an approximate temperature of 4°C, and they were transported to the Microbiology Teaching Laboratory at the College of Medicine and Health Science, University of Gondar within 3 hours. In the laboratory, the specimens were inoculated onto modified cefoperazone charcoal deoxycholate agar (Oxoid). The inoculated plates were subsequently placed in an anaerobic jar where a microaerophilic environment (to 5% O_2_, 10% CO_2_, and 85% N_2_) was adjusted using gas-generating sachets (Oxoid), and they were incubated at 42°C for 48 hours.^21^

### Isolation and identification of *Campylobacter* species.

After a 48-hour incubation, the suspected growth was examined macroscopically for gray color, moist spreading, and metallic sheen colonies. Microscopic examination was conducted to detect the darting movement and Gram-negative reaction, with particular attention to the colonies’ curved and S-shaped structures. Catalase and oxidase tests were performed to determine the presence of these enzymes in the suspected colonies. Subsequently, the suspected colonies were inoculated onto Colombia blood agar (Oxoid) and incubated in a microaerophilic environment at 37°C for 24–48 hours.^21^ The colonies grown on blood agar were analyzed utilizing matrix-assisted laser desorption ionization–time-of-flight mass spectrometry (MALDI-TOF MS) from Bruker Daltonics (Bremen, Germany). For the preparation of samples, a thin layer of each isolate’s colony segment was applied to a ground steel plate using a sample applicator and allowed to air dry. Following this, 1 µL of in vitro diagnostic (IVD) bacterial test standard (BTS) was introduced to each designated area and allowed to dry at ambient temperature. Subsequently, 1 µL of IVD α-Cyano-4-hydroxycinnamic acid (HCCA) matrix solution was added on top of the sample and IVD BTS, and it was then allowed to crystallize at room temperature. The matrix-assisted laser desorption ionization target plate was then placed into the mass spectrometer. Measurements were performed using the Ultraflex III time-of-flight/time-of-flight mass spectrometer equipped with a 200-Hz smart beam 1 laser from Bruker Daltonics. A control sample of *Escherichia coli*, provided by Bruker Daltonics, was included in each run to verify the operational integrity of the spectrometer. The raw spectra of the strains were analyzed using the MALDI BIOTYPER 2.0 software. The entire procedure from the MALDI-TOF MS measurements to the identification phase was carried out automatically without any user intervention.

### Antimicrobial susceptibility test.

Upon confirmation of *Campylobacter* species, the Kirby–Bauer modified disc diffusion technique was performed on BD Mueller Hinton fastidious agar (MH-F) following the European Committee on Antimicrobial Susceptibility Testing (EUCAST) guidelines.^22^ Approximately two to three pure colonies were suspended in sterile normal saline and adjusted to 0.5 McFarland standard using a McFarland densitometer (Grant-bio, Cambridgeshire, England). A sterile cotton swab was dipped into the suspension and gently squeezed against the inside wall of the test tube to prevent heavy inoculation. The strain was then evenly spread on the entire BD MH-F plate. The inoculated plates were allowed to air dry for 3–5 minutes. Following EUCAST recommendations, ciprofloxacin (5 *µ*g), erythromycin (15 *µ*g), and tetracycline (30 *µ*g) discs were placed on the MH-F plate using a disc dispenser. The plates were then placed in an anaerobic jar with gas-generating sachets and incubated at 42°C for 24 hours. After the 24-hour incubation period, the diameter of the zone of inhibition was measured using a ruler. Multidrug-resistant strains were identified based on previously established criteria.^23^

### Data quality assurance.

Trained laboratory professionals were recruited for interviews and sample collection. A standard operational procedure was followed during any laboratory analysis. To ensure sterility, a test was conducted on 5% of the prepared media by incubating it for 1–2 days depending on the type of media. Additionally, the quality of antimicrobial discs was assessed using control strains (*C. jejuni* NCTC 11351 and *C. coli* NCTC 11351). The proper functionality of the MALDI-TOF MS assay was verified using the BTS. The overall quality of the laboratory was maintained by adhering to preanalytical, analytical, and postanalytical quality control measures.

## STATISTICAL ANALYSES

After ensuring the completeness of the data, they were entered into the SPSS v. 25 statistical package (IBM Corp., Armonk, NY) for analysis. The analysis included crosstabulation and frequency tables using SPSS. The Fisher exact test was applied to identify potential factors linked to antibiotic-resistant *Campylobacter* strains. Any *P*-value below 0.05 within a 95% CI was considered statistically significant.

## RESULTS

### The reproduction condition of the free-range chickens.

A total of 286 households that kept free-range chickens participated in this study. On average, each household had 4.7 chickens, with a range of 1–20 chickens per household.

Nearly equal numbers of pooled samples of free-range chickens were collected from the study sites (Gondar = 150 and Bahr Dar = 136). Of the total samples, 53.5% were collected from chickens living with a variety of animals. One third of the owners were using drugs to promote the growth of their chickens. In addition, a quarter of the owners had used drugs for treatment purposes. The majority (57.0%) of the owners were living with their chickens in the same house (Supplemental Table 1). The results of the binary logistic regression analysis indicated that the prevalence of *Campylobacter* species was not significantly associated with the number of chickens, cohabitation with other animals, the use of drugs for growth promotion, the use of drugs for treatment, or the practice of shared housing (*P* >0.05).

### Prevalence of *Campylobacter*.

In this study, a total of 286 pooled cloacal swabs were collected from free-range chickens. Among these samples, 43 were found to be positive for *Campylobacter* species. The overall prevalence of *Campylobacter* was determined to be 15.0% (CI: 10.2–19.5), with *C. jejuni* and *C. coli* having prevalence rates of 9.1% and 5.9%, respectively. *C. jejuni* was more prevalent, accounting for 60.5% of the positive samples, whereas *C. coli* accounted for 39.5% (Supplemental Table 2).

### Antimicrobial resistance of *Campylobacter*.

**Figure 1.**
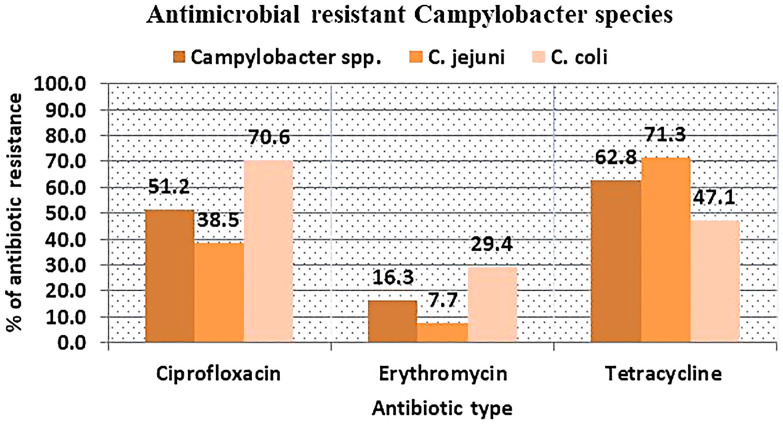
Antimicrobial-resistant *Campylobacter* species among free-range chickens in Amhara Region, northwest Ethiopia from November 2022 to April 2023.

Forty-three confirmed *Campylobacter* species were subjected to antimicrobial susceptibility testing using ciprofloxacin, erythromycin, and tetracycline discs. The findings revealed that 62.8% (*n* = 27/43), 51.2% (*n* = 22/43), and 16.3% (*n* = 7/43) of the *Campylobacter* species displayed resistance to tetracycline, ciprofloxacin, and erythromycin, respectively. Among *C. coli* strains, 70.6%, 47.1%, and 29.4% were resistant to ciprofloxacin, tetracycline, and erythromycin, respectively (Figure 1).

### Potential factors associated with ciprofloxacin, erythromycin, and tetracycline resistance in *Campylobacter* species.

The Fisher exact test was used to assess the association between potential factors and the resistance level of *Campylobacter* species to ciprofloxacin, erythromycin, and tetracycline. As a result, the resistance levels of *Campylobacter* species to ciprofloxacin and erythromycin had no association with factors. However, having mixed animals with chickens (*P* = 0.01) and the use of the drug for treatment (*P* = 0.01) were significantly associated with the resistance level of *Campylobacter* species to tetracycline (Supplemental Table 3).

### Multiple and multidrug resistance.

Table 1 presents the antimicrobial resistance profile of the *Campylobacter* species identified, showing resistance to macrolides (erythromycin), fluoroquinolones (ciprofloxacin), and tetracycline. Among the total isolates, 62.8% (*n* = 27/43) exhibited resistance to two or more antibiotics. The most common resistant patterns were ciprofloxacin–tetracycline (*n* = 16/43; 37.2%) followed by ciprofloxacin–erythromycin (*n* = 7/43; 16.3%), and erythromycin–tetracycline (*n* = 4/43; 9.3%). Additionally, the resistance rates to ciprofloxacin–erythromycin (29.4%) and ciprofloxacin–tetracycline (41.2%) were higher in *C. coli* compared with *C. jejuni*. Four of 43 strains (9.3%) were identified as multidrug-resistant (ciprofloxacin–erythromycin–tetracycline) *Campylobacter* species.

**Table 1:** Distribution of multidrug-resistant profiles of *Campylobacter jejuni* and *Campylobacter coli* among free-range chickens in Amhara Region, northwest Ethiopia from November 2022 to April 2023

Antimicrobial Pattern	*Campylobacter* spp. (*n* = 43)	*Campylobacter jejuji* (*n* = 26)	*Campylobacter coli* (*n* = 17)
CIP^R^-ERY^R^	7 (16.3%)	2 (7.7%)	5 (29.4%)
CIP^R^-TET^R^	16 (37.2%)	9 (34.6%)	7 (41.2%)
ERY^R^-TET^R^	4 (9.3%)	2 (7.7%)	2 (11.8%)
ERY^R^-CIP^R^-TET^R^	4 (9.3%)	2 (7.7%)	2 (11.8%)

CIP^R^-ERY^R^ = ciprofloxacin–erythromycin resistant; CIP^R^-TET^R^ = ciprofloxacin–tetracycline resistant; ERY^R^-CIP^R^-TET^R^ = erythromycin–ciprofloxacin–tetracycline resistant; ERY^R^-TET^R^ = erythromycin–tetracycline resistant.

## DISCUSSION

Chicken production makes a remarkable contribution to food security, employment, and economic development in urban and rural parts of African countries.^24^^,^^25^ In contrast, chickens can be a potential source of different types of infectious diseases.^26^ The present study aimed to see the prevalence and antimicrobial resistance of *Campylobacter* species among free-range chickens in Amhara National Regional State, northwest Ethiopia.

In the current investigation, the overall occurrence of *Campylobacter* species among free-range chickens was recorded at 15.0%. This result aligns closely with the research conducted by Chala et al.^27^ in Ethiopia, which reported a prevalence of 13.0%, and Suman Kumar et al.^28^ in India, who found a prevalence of 16.8%. Conversely, a previous study by Acsa et al.^29^ in Kenya reported a lower prevalence of 2.5%. Nevertheless, our study’s prevalence is lower than in various studies conducted worldwide, including those conducted in northern Tunisia (22.4%)^30^; Iran (25.0%)^31^; eastern China (32.4%)^32^; Korea (35.9%)^33^; Italy (43.5%)^34^; Shanghai, China (57.4%)^35^; Japan (75.1%)^36^; and Turkey (82.3%).^37^

Variations in the prevalence of *Campylobacter* species among chickens in different studies may be because of factors such as poultry production systems, study seasons, inadequate biosafety measures, and geographical and climatic conditions as well as the laboratory methods used. This particular study was conducted during a dry season (November to April) when a lower prevalence of *Campylobacter* species was expected. Some research findings have shown that the occurrence of *Campylobacter* species in chickens tends to be lower during dry seasons.^38^^,^^39^ Furthermore, our study focused solely on *C. jejuni* and *C. coli* unlike previous studies that included other species, which could impact the prevalence of *Campylobacter* species.^27^ Prachantasena et al.^40^ found a significant association between *Campylobacter* colonization in chickens, humidity, rainfall. In addition to conventional culture and biochemical testing, our study also used matrix-assisted laser desorption ionization–time-of-flight technology, which is known for its high specificity and sensitivity compared with culture and polymerase chain reaction methods, potentially contributing to the observed variations in *Campylobacter* species prevalence.^41^^,^^42^ A study from Portugal also suggested that different production systems can influence the variation in *Campylobacter* colonization in chickens.^43^

Our research also demonstrated that *C. jejuni* (60.5%) had a higher frequency compared with *C. coli* (39.5%). This finding aligns with a previous study conducted by Wayou et al.^44^ in central Ethiopia and other studies conducted in various regions worldwide, including Benin,^45^ Kenya,^46^ Côte d’Ivoire,^47^ Tunisia,^30^ Mongolia,^48^ Thailand,^49^ Spain,^50^ and Canada.^51^ The increased presence of *C. jejuni* in free-range chickens may be attributed to its ability to survive in the environment for extended periods when compared with *C. coli*. Nilsson et al.^52^ noted that *C. jejuni* exhibited better survival rates than *C. coli* in water environments. Additionally, Bronowski et al.^53^ highlighted that *C. jejuni* can persist through various mechanisms, such as aerotolerance, resistance to starvation, biofilm formation, and transitioning to a nonculturable viable form, thereby enhancing the likelihood of frequent detection.

In the present investigation, it was observed that approximately two thirds (62.8%) of the confirmed *Campylobacter* species displayed resistance to tetracycline. The prevalence of tetracycline-resistant *Campylobacter* species in free-range chickens can potentially be attributed to the irrational utilization of tetracycline for both treatment and growth promotion in chicken production. Furthermore, these free-range backyard chickens may come into contact with fecal matter containing tetracycline-resistant *Campylobacter* while foraging in their surroundings. Our research findings regarding resistance rates align with a study conducted in Spain, which reported a similar rate of 65%.^50^ However, previous studies conducted in Ethiopia documented lower resistance rates.^27^^,^^54^ Similarly, other countries worldwide have reported lower prevalence rates, such as Italy with a rate of 39.7%^34^ and India with a rate of 48.6%.^28^

Furthermore, our investigation also identified that 51.2% of the *Campylobacter* species exhibited resistance to ciprofloxacin, which is alarming considering its role as an alternative treatment of gastroenteritis. This finding indicates a relatively higher resistance rate compared with reports from various countries, such as northwest South Africa (19.2%),^55^ Italy (26.6%),^34^ and India (32.9%).^31^ Conversely, studies conducted by Kouglenou et al.^45^ in Benin (72.7%), Chala et al.^27^ in Ethiopia (77.8%), Marin et al.^50^ in Spain (95.0%), and Gharbi et al.^30^ (99.2%) reported even higher rates of ciprofloxacin resistance. The high rate of ciprofloxacin resistance in our study may be attributed to the prophylactic (growth promotion) and therapeutic use of fluoroquinolones in poultry production or the transmission of ciprofloxacin-resistant strains from humans to chickens.^56^^,^^57^

In this research, a relatively low rate of erythromycin resistance (16.3%) was observed, which is similar to the findings from Benin (16.5%).^45^ However, the current study reports a significantly lower resistance rate compared with a previous study conducted in Ethiopia (53.0%).^54^ The difference in resistance rates could potentially be attributed to variations in the production systems. The present study specifically focused on organic poultry production, whereas the previous study examined conventional production systems where antibiotics, such as tylosin, may have been used in chicken feed.^58^ The relatively low level of erythromycin resistance suggests that this antibiotic could be considered as a preferred treatment option for human *Campylobacter* gastroenteritis.

Our study identified that having other animals with chickens and using drugs for treatment of chickens were associated with tetracycline-resistant *Campylobacter* species. Cohabitation or rearing of chickens with other domestic animals that have been colonized with tetracycline-resistant *Campylobacter* strains might pose a potential risk for transmission of these resistant strains. In addition, irrational antimicrobial uses, like tetracycline for the treatment of free-range chickens, might be associated with the development of tetracycline-resistant *Campylobacter* species.^59^

The prevalence of multidrug-resistant *Campylobacter* species in free-range chickens was found to be 9.3%, which is a cause for concern. The development of multidrug resistance in *Campylobacter* strains can be attributed to the acquisition of various resistance genes on a single DNA molecule or through the action of a single-efflux pump mechanism that expels multiple antibiotics from bacterial cells. In the case of *C. jejuni* and *C. coli*, the multidrug resistance efflux pump known as campylobacter multidrug efflux system A-B-C is responsible for expelling ciprofloxacin (a fluoroquinolone), erythromycin (a macrolide), and tetracycline. The current finding is comparable with previous studies conducted in Côte d’Ivoire (9.4%),^47^ Ethiopia (14.1%),^27^ and northwest South Africa (15.0%).^55^ However, other African countries reported higher findings, such as Benin (55.8%),^45^ Kenya (61.3%),^46^ and northern Tunisia (100%).^30^ On the other hand, lower levels were documented in Ghana (4.7%).^39^ Discrepancies in multidrug resistance rates reported by different researchers may be because of variations in the selection of antibiotic discs for antimicrobial susceptibility testing. Our isolates were tested against ciprofloxacin, erythromycin, and tetracycline as recommended by EUCAST.

The current study has certain limitations. First, the sampling was restricted to northwest Ethiopia, which means that the data used in the study may not accurately represent the entire country because of geographical variations that can influence the occurrence and antimicrobial resistance profiles of *Campylobacter* species. Moreover, our study was conducted during the dry season, which could potentially impact the prevalence of *Campylobacter* species. The prevalence might be influenced by factors such as humidity and rainfall. Additionally, we encountered a loss of a few presumptive *Campylobacter* strains during the storage period in deep freeze.

## CONCLUSION

The prevalence of *Campylobacter* species among free-range chickens was documented to be 15.0%. Notably, there was a significant proportion of tetracycline- and ciprofloxacin-resistant *Campylobacter* species. Tetracycline resistance was more prevalent in *C. jejuni*, whereas *C. coli* exhibited a higher prevalence of ciprofloxacin resistance. The presence of multidrug-resistant *Campylobacter* species in this study highlights the importance of taking special measures to prevent the potential transmission of these strains to the wider human community. It is crucial to raise awareness among agricultural sector offices and poultry owners regarding the appropriate use of antimicrobial agents for free-range chickens. Additionally, it is recommended to expand the study to adopt a One Health approach, conducting nationwide research to gain a comprehensive understanding of the distribution of *Campylobacter* species and their patterns of antimicrobial resistance.

## Supplemental Materials

10.4269/ajtmh.24-0578 Supplemental Materials
