# Supplementary material for: Prevalence and Antimicrobial-Resistant Campylobacter jejuni and Campylobacter coli in Free-Range Chickens in Northwest Ethiopia
Source: Am J Trop Med Hyg. 2025 Jun 24;113(3):694–700. doi: 10.4269/ajtmh.24-0578 (PMC12410166; doi:10.4269/ajtmh.24-0578)
Supplement: Supplemental Materials [file tpmd240578.SD1.pdf]

Tables

Supplemental file 1: The reproduction system of free-range backyard chickens in the Amhara National Region state, Northwest Ethiopia, November, 2022 to April, 2023

**Variables**	**Category**	**Frequency (%)**
Study site	Gondar	150 (52.4)
	Bahir Dar	136 (47.6)
Owing of mixed animal	Yes	153 (53.5)
	No	133 (46.5)
Using of drug for growth promotion	Yes	83 (29.1)
	No	203 (71.0)
Using drug for treatment	Yes	72 (25.2)
	No	214 (74.8)
Chickens sharing the same residence with owners	Yes	123 (57.0)
	No	163 (43.0)
Campylobacter species	Positive	43 (15.0)
	Negative	243 (85.0)

Supplemental file 2: The overall prevalence of *Campylobacter* species among free-range backyard chicken in the Amhara National Region state, Northwest Ethiopia, November, 2022 to April, 2023

**Strain**	***Campylobacter* infection**	
	**Positive (%)**	**Negative (%)**
Campylobacter species	43 (15.0%)	243 (85.0)
• *Campylobacter jejun*	26 (9.1%)	260 (90.9)
• *Campylobacter coli*	17 (5.9 %)	269 (94.1)

Supplemental file 3: **Factors associated with *Campylobacte*r resistance to tetracycline in free-range backyard chicken in Amahara National Regional state, Northwest Ethiopia, November, 2022 to April, 2023**

**Predictors**	**Tetracycline**		***P value***
	**Sensitive (%)**	**Resistant (%)**	
**Having of mixed animal**			0.01*
No	11	7	
Yes	5	20	
**Use of drug for growth**			0.72
No	13	20	
Yes	3	7	
**Use of drug for treatment**			0.01*
No	11	7	
Yes	5	20	
**Shared house with human**			0.13
No	6	17	
Yes	10	10	
**Species type**			0.11
C. coli	9	8	
C. jejuni	7	19	

* Statistically significant
